# Effects of a 6-Week High-Intensity Interval Training on Physical Fitness in Female Basketball Players: A Randomized Controlled Trial

**DOI:** 10.3390/jfmk11030271

**Published:** 2026-07-14

**Authors:** Ilma Čaprić, Ivana Bojić, Miodrag Kocić, Omer Špirtović, Raid Mekić, Adem Mavrić, Luka Pezelj, Igor Jelaska, Mima Stanković

**Affiliations:** 1 Department of Biomedical Sciences, State University of Novi Pazar, 36300 Novi Pazar, Serbia; icapric@np.ac.rs (I.Č.); ospirtovic@np.ac.rs (O.Š.); rmekic@np.ac.rs (R.M.); amavric@np.ac.rs (A.M.); 2 Faculty of Sport and Physical Education, University of Niš, 18000 Niš, Serbia; ivana.bojic@fsfv.ni.ac.rs (I.B.);; 3 Faculty of Maritime Studies, University of Split, 21000 Split, Croatia; luka.pezelj@pfst.hr; 4 Faculty of Kinesiology, University of Split, 21000 Split, Croatia; jelaska@kifst.hr

**Keywords:** HIIT, female basketball players, agility, sprint performance, aerobic capacity, repeated sprint ability, explosive power

## Abstract

**Objectives:** High-intensity interval training (HIIT) has become an increasingly popular conditioning strategy in women’s basketball due to its effectiveness in improving physical performance. The aim of this study was to examine the effects of a six-week HIIT on combined aerobic and anaerobic capacity, running speed, agility, repeated sprint ability, and explosive power in elite female basketball players. **Methods:** Thirty elite female basketball players (21.81 ± 2.12 years) were randomly assigned to either a HIIT group (*n* = 15) or a control group (*n* = 15). **Results:** Pre- and post-intervention assessments included running speed (0–20 m); the 5–0–5, Zig-zag, and Pro-agility tests; vertical jump performance (CMJ, CMJA, and SJ); repeated sprint ability (RSA); aerobic capacity assessed by the 30–15 Intermittent Fitness Test; and VO_2_max. Statistically significant Group × Time interaction effects emerged for running speed (5 m, 10 m, and 20 m), aerobic capacity (30–15 IFT and VO_2_max), and Zig-zag agility performance (*p* < 0.05), indicating greater improvements in the HIIT group compared with the control group. Significant main effects of time were observed for RSA and vertical jump performance, indicating improvements in both groups; however, no significant Group × Time interactions were found. **Conclusions:** These findings suggest that a six-week HIIT is an effective and time-efficient conditioning strategy for enhancing aerobic capacity, sprint performance, and change-of-direction ability in elite female basketball players.

## 1. Introduction

Women’s basketball is a high-intensity intermittent sport characterized by frequent accelerations and decelerations, repeated sprint actions, rapid changes of direction, jumping activities, and explosive movements performed under conditions of continuous technical and tactical demands [1,2]. Owing to the intermittent, multidirectional, and metabolically demanding nature, female basketball players are required to possess well-developed aerobic power, anaerobic glycolytic capacity, and neuromuscular efficiency, together with sport-specific motor abilities, in order to maintain optimal performance throughout the entire match [3,4,5]. Modern competitive basketball additionally imposes substantial physiological and neuromuscular demands on athletes, emphasizing the importance of optimal physical preparation and the implementation of effective conditioning strategies in female basketball players [2,6,7,8]. High-intensity interval training (HIIT) has gained prominence as a conditioning modality in women’s basketball due to the growing physical demands of the sport and the need for simultaneous development of aerobic and anaerobic capacities, explosive power, agility, and sport-specific motor skills [5,9,10,11,12,13]. In women’s basketball, these demands are further emphasized by frequent high-intensity transitions during offensive and defensive phases, repeated involvement in accelerations and decelerations across all playing positions, and the necessity to maintain performance across congested match schedules, making HIIT particularly relevant for the simultaneous development of aerobic and neuromuscular capacities in this population [4]. Furthermore, different HIIT modalities, including sprint interval training, neuromuscular HIIT, and change-of-direction HIIT, have demonstrated positive effects on physiological and neuromuscular adaptations, with some studies reporting improvements comparable or even superior to those achieved through plyometric training or small-sided games [11,12]. Short sprint interval training has also been shown to significantly improve vertical jump performance, explosive strength, agility, sprint performance, and VO_2_max in female basketball players, while sex did not significantly influence the magnitude of adaptation [5]. In addition to enhancing physical performance, HIIT has been identified as a time-efficient training strategy capable of producing meaningful adaptations during relatively short preparatory periods, which is particularly important during congested competitive schedules [9,10,12,13]. Although the findings are generally positive, some studies emphasize the need for individualized HIIT protocols and their combination with sport-specific technical training in order to optimize all aspects of basketball performance [12,13]. Recent evidence in women’s team-sport athletes suggests that HIIT induces concurrent improvements in aerobic capacity, repeated sprint ability, and neuromuscular performance when applied in sport-specific training environments [14].

Despite the growing body of evidence supporting the effectiveness of HIIT in basketball conditioning, the evidence base specific to female basketball players remains scarce. In accordance with the participant classification framework proposed by McKay et al. (2021) [15], the term ‘elite’ is operationally defined as Tier 3 athletes, representing highly trained, national-level competitors, and was used in this study to describe female basketball players competing at the highest national league level. However, most previous studies have examined the effects of HIIT on one or two isolated performance variables, whereas the present study aimed to simultaneously investigate a broader set of outcomes, including aerobic–anaerobic fitness, sprint performance, agility, repeated sprint ability, and explosive power in elite female basketball players. To the best of the authors’ knowledge, no previous study has investigated the effects of sport-specific HIIT on multiple physical performance parameters in elite female basketball players at the national competitive level. Studying this population is particularly relevant from both a scientific and applied perspective, given the high physical and competitive demands of elite women’s basketball and the need for evidence-based training strategies to optimize performance in high-level athletes. Accordingly, the aim of this study was to examine the effects of high-intensity interval training on aerobic–anaerobic fitness, sprint performance, agility, repeated sprint ability, and explosive power in female basketball players, with particular emphasis on the practical implications for the training process and future research in this field. It was hypothesized that the HIIT intervention would result in significant improvements in measures of aerobic fitness (VO_2_max and VIFT), sprint performance over short and moderate distances (5 m, 10 m, and 20 m), change-of-direction ability (Zig-zag and 5–0–5 tests), repeated sprint ability, and lower-limb explosive power in female basketball players, compared with a control group.

## 2. Materials and Methods

### 2.1. Participants

Sample size estimation was performed using G*Power software version 3.1 (University of Bonn, Bonn, Germany). Assuming a significance level of α = 0.05, statistical power of 1 − β = 0.80, and a medium effect size (f = 0.25), the analysis indicated that a minimum of 27 to 32 participants was required to identify statistically significant effects for a repeated-measures design. The study included thirty elite female basketball players competing at the highest national level (age: 21.81 ± 2.12 years; body height: 176.04 ± 3.32 cm; body mass: 69.84 ± 10.10 kg). Following baseline testing, using a computer-generated randomization sequence, participants were randomly allocated into either the HIIT group (*n* = 15; age: 23.31 ± 4.19 years; body height: 178.38 ± 6.82 cm; body mass: 69.13 ± 9.28 kg; training experience: 6.3 ± 4.4 years) or the control group (CON) (*n* = 15; age: 20.31 ± 2.50 years; body height: 173.69 ± 6.25 cm; body mass: 70.55 ± 10.92 kg; training experience: 6.9 ± 5.2 years). The study employed a parallel-group design in which the experimental group performed a six-week HIIT program in addition to regular basketball training, while the control group continued their usual training routine. The detailed HIIT protocol is presented in Section 2.4.

From an initial sample of 30 participants, 30 athletes met the inclusion criteria and were randomly allocated to the HIIT group (*n* = 15) and the control group (*n* = 15). An 80% attendance rate was a requirement for inclusion in the study, and all participants met this criterion. Therefore, the final analysis included 15 participants in the HIIT group and 15 participants in the control group. Group allocation was carried out using a computer-generated randomization procedure, stratified according to playing position in order to maintain balanced group characteristics. The randomization process was conducted by an independent researcher, while group assignments remained concealed from the investigators responsible for training implementation and performance testing, following the recommendations proposed by Schulz [16]. The randomization sequence was generated by an independent researcher, and allocation was concealed until group assignment. Both groups continued their regular basketball training with comparable weekly training volume, including standard conditioning, technical, and tactical sessions.

Eligibility criteria required participants to be aged ≥16 years, actively competing at the highest level of basketball competition, and regularly participating in at least five training sessions per week. Participants were excluded if they had cardiovascular, respiratory, or other medical conditions; were currently undergoing rehabilitation; had sustained an injury within the previous three months; or had undergone anterior cruciate ligament (ACL) reconstruction surgery within the last year. Prior to participation, all athletes aged 18 years or older provided written informed consent. For participants younger than 18 years, written informed consent was obtained from their legal guardians. Ethical approval for the study was granted by the Ethics Committee of the Faculty of Sport and Physical Education, University of Niš, and all procedures were conducted in accordance with the Declaration of Helsinki (Ref. No. 04–92/2, date of approval 21 January 2026). Additionally, the club management and all participants were fully informed about the purpose and procedures of the investigation.

### 2.2. Procedures

The assessment of physical performance in all participants at baseline and final testing was conducted in a multidisciplinary diagnostic center as well as in a sports hall, and the entire testing process lasted two days. All measurements on both days were performed within the time interval from 9:00 to 11:00 a.m. The order of tests was identical at the initial and final assessments. In most cases, results were automatically recorded through direct data transfer from the measuring instruments to a computer, whereas in situations where software solutions were unavailable, data were entered manually into previously prepared recording forms. Before testing, all participants completed a standardized 20 min warm-up protocol consisting of light running (6 min), stretching exercises (5 min), progressive acceleration running (3 min), change-of-direction (COD) drills (3 min), and low-intensity plyometric jumps (3 min). After the warm-up, vertical jump tests (CMJ, countermovement jump with arm swing—CMJA, and squat jump—SJ), as well as the repeated sprint ability (RSA) test, were performed. On the second day, following the identical warm-up protocol, participants completed additional physical performance tests, including a 20 m sprint with split times at 5 m and 10 m, the Pro-agility test, the Zig-zag test, and the 30–15 Intermittent Fitness Test (30–15 IFT). All tests, as well as the complete measurement procedure, were conducted in the same order and under identical conditions during both baseline and final testing for all participants. Post-intervention testing was performed within one week following completion of the six-week intervention. Participants were instructed to refrain from strenuous exercise for at least 48 h before each testing session and to maintain their usual dietary and hydration habits.

### 2.3. Physical Performance Assessment

#### 2.3.1. Speed (Running 0–20 m)

Linear sprint tests over distances of 5 m, 10 m, and 20 m were used to assess sprint speed. Time measurement was performed using three pairs of infrared photocells (Microgate, Polifemo Radio Light, Bolzano, Italy). Prior to testing, participants were thoroughly familiarized with the testing protocol. The starting position was marked 30 cm behind the first pair of photocells. Participants were instructed to sprint maximally over the 20 m distance from a stationary position. Timing was automatically triggered upon passing through the start gate, with split times recorded at 5 m and 10 m. Acceleration performance was evaluated based on the time required to cover the first 5 m. Each participant performed three maximal attempts with a minimum rest interval of 3 min between trials. The best result achieved was used for further statistical analysis [17,18].

#### 2.3.2. 5–0–5 Test (Agility)

Agility involving a 180° change of direction was assessed using the 5–0–5 test. The test was conducted on an indoor basketball court, and time was measured using photocell timing gates positioned at hip height, with 120 cm between photocell stations. One pair of photocells was placed at the starting line (A), the second at a distance of 15 m (C), and the third at the finish line (B). Participants assumed a standing start position at line A. Upon the auditory signal, they sprinted maximally to point C, executed a 180° turn, and continued sprinting maximally to the finish line B. Each participant performed six attempts in total (three with the right leg and three with the left leg), with at least 3 min of rest between attempts. The final result was calculated as the mean value of the three trials for each leg, expressed in seconds, and represented the overall agility score for each direction of movement change [19].

#### 2.3.3. Zig-Zag Agility Test

Running agility involving multiple changes of direction was assessed using the Zig-zag test. The test course consisted of four 5 m sections (total distance 20 m), with cones positioned at 100° angles, using two pairs of photocells (Witty, Microgate, Bolzano, Italy). Participants were required to decelerate, change direction, and re-accelerate around each cone as quickly as possible. The test began from a standing position, with the front foot positioned 30 cm behind the first pair of photocells. The task was to complete the course at maximal speed until crossing the finish gate. Each participant performed up to three attempts, with at least 3 min of rest between trials. This test has previously been applied in studies involving athletes competing at the elite level [18].

#### 2.3.4. Pro-Agility Test (5–10–5 m)

The ability to rapidly change direction in the lateral plane was assessed using the Pro-agility test (5–10–5 m). Time measurement was performed using photocell timing systems (Microgate, Polifemo Radio Light, Bolzano, Italy). Participants started from a central position between two cones placed 10 m apart. The timing system was activated by passing through the start gate. In the first attempt, participants independently selected the movement direction (left or right), while the opposite direction was used in the subsequent attempt. The task consisted of a lateral sprint to the first cone positioned 5 m away, followed by a sprint in the opposite direction to the cone positioned 10 m away, and finally returning to the starting position (5 m). Timing ended upon re-crossing the start line. Each participant performed three attempts, with at least 3 min of passive recovery between trials. The best achieved result was used for analysis. The reliability and validity of this test have been confirmed in previous studies [19].

#### 2.3.5. Vertical Jumps (CMJ, CMJA, SJ)

Vertical jump height (cm) was assessed using three standardized tests: countermovement jump (CMJ), countermovement jump with arm swing (CMJA), and squat jump (SJ). Flight time, from which jump height was calculated, was measured using the Optojump photoelectric system (Microgate, Bolzano, Italy). Prior to testing, participants were familiarized with the proper execution technique and instructed to achieve maximal jump height. During the CMJ test, participants performed the jump with their hands placed on their hips, following a preparatory knee flexion of approximately 90°, upon the given signal, without removing their hands from the body. The CMJA test was performed according to the same procedure as the CMJ, except that a free arm swing was allowed throughout the movement. The squat jump (SJ) was executed from a static starting position with knees flexed at a 90° angle and hands on the hips, maintained for approximately 3 s before take-off. During the SJ test, additional countermovement of the knees or trunk was not permitted. Each test consisted of three attempts, with 1 min of passive recovery between trials and 3 min of rest between different tests. The best achieved result, expressed in centimeters, was used for further analysis. The validity and reliability of all listed tests have been confirmed in previous studies [20].

#### 2.3.6. Repeated Sprint Ability (RSA)

Repeated sprint ability was assessed using the RSA test, consisting of six 40 m sprints with 20 s of passive recovery between each sprint. Timing was measured using infrared photocell timing gates (Microgate, Polifemo Radio Light, Bolzano, Italy). Two pairs of photocells were positioned at the starting line, while an additional two pairs were placed in a straight line 20 m from the start. Participants began the test from a position 30 cm behind the starting line. Upon the readiness signal, participants passed through the start gate and sprinted maximally to the marked line 20 m away, where they performed a rapid turn and sprinted back toward the starting line. Timing automatically stopped upon re-crossing the start gate. Before each sprint, participants were verbally instructed to assume the starting position 30 cm behind the line, with a three-second countdown before restarting. This procedure was repeated six times, with 20 s of recovery between repetitions. Two performance indicators were calculated for each participant: average sprint time (RSAavg) and total sprint time (RSAtime), expressed in seconds with a precision of 1/100. Considering the progressive fatigue during the test, a fatigue index (FI) was additionally determined, calculated as the relative difference between the best and worst sprint time and expressed as a percentage. The validity and reliability of the RSA test and its associated indicators have been confirmed in previous studies [21].

#### 2.3.7. 30–15 Intermittent Fitness Test

VO_2_max was estimated from the 30–15 Intermittent Fitness Test. The 30–15 Intermittent Fitness Test was conducted on a 40 m court with cones positioned at the starting and finishing lines, while two 3 m zones were marked in the central part of the test course to provide visual guidance for maintaining the prescribed running speed. Each participant ran back and forth between the two lines while following the pace determined by an auditory signal. The initial running speed was set at 8 km/h and maintained for 30 s, after which the speed increased by 0.5 km/h at each subsequent stage (every 45 s). During the 15 s active recovery period, participants walked toward the nearest line depending on their position at the end of the previous stage. Throughout the test, participants were verbally encouraged to achieve maximal performance and cover the greatest possible total distance. The test was terminated when the participant could no longer maintain the required running speed or failed to reach the 3 m zone around the line at the moment of the auditory signal during three consecutive attempts. Based on the 30–15 IFT results, maximal oxygen uptake was indirectly estimated and expressed in relative and absolute values. VO_2_max values were estimated indirectly from performance in the 30–15 IFT using validated prediction equations, rather than being directly measured via laboratory-based gas analysis. The final completed speed at the last stage of the 30–15 IFT was defined as VIFT (final intermittent running speed), which was used for training prescription and analysis. The validity and reliability of this test have been confirmed in previous studies [17].

### 2.4. Training Interventions

HIIT was conducted during the preparatory phase of the season and lasted six weeks, with training sessions performed twice per week (Tuesday and Thursday). All training sessions were performed under similar environmental conditions and at the same time of day to minimize external variability. Throughout the six-week intervention, both groups participated in the same regular preseason basketball training program. Team practices were conducted five times per week and lasted approximately 90–100 min per session. Each training session began with a standardized 15 min warm-up consisting of low-intensity running, dynamic mobility exercises, and basketball-specific movement preparation. The main part of the session (65–75 min) included technical drills (shooting, passing, dribbling, footwork, and individual offensive and defensive skills), tactical training focused on offensive and defensive systems, transition offense and defense, positional play, and controlled game situations. Training sessions concluded with a 10 min cool-down involving low-intensity running and static stretching. The overall training content, duration, and coaching staff were identical for both groups throughout the intervention. The only difference between groups was that the experimental group performed the prescribed HIIT protocol twice weekly (Tuesday and Thursday) instead of the standard conditioning component of the regular basketball practice, whereas the control group completed the standard conditioning activities as planned by the coaching staff. All HIIT sessions were supervised by qualified strength and conditioning coaches to ensure adherence to the prescribed intensity and correct execution of the protocol. To ensure adequate recovery, a minimum rest interval of 48 h was maintained between HIIT sessions. Each training session started with a standardized warm-up lasting approximately 15 min. The warm-up consisted of moderate-intensity running (4 min), a combination of static and dynamic stretching exercises (5 min), and passing and ball reception drills (4 min), as well as short sprint and acceleration activities (2 min). Preparatory exercises targeting the activation of muscle groups primarily involved in the main training segment were also incorporated, including lateral jumps, eccentrically controlled lunges, trunk stabilization exercises, and agility drills involving changes of direction. Upon completion of the main training segment, a cool-down period of approximately 10 min was performed, consisting of light jogging and static stretching of the major muscle groups. The protocol primarily consisted of high-intensity running-based intermittent efforts with a repeated acceleration–deceleration pattern. The main part of the HIIT protocol (Table 1) was based on interval running performed at an intensity corresponding to 100% of the individually determined VIFT achieved during the 30–15 Intermittent Fitness Test (VIFT). Work and recovery intervals were organized according to a 15 s work-to-15 s passive recovery ratio. The protocol was designed to replicate the intermittent and multidirectional movement patterns typical of basketball match play. Training load was progressively increased throughout the intervention period. During weeks 1 and 2, participants completed three sets of eight intervals; during weeks 3 and 4, three sets of ten intervals were performed, whereas during weeks 5 and 6, participants completed three sets of twelve intervals. The rest interval between sets lasted 3 min [22].

The applied HIIT protocol was specifically designed to include a mandatory 180° change of direction during every running interval, thereby increasing both metabolic and neuromuscular stress during exercise. Following the recommendations proposed by Laursen and Buchheit [22], the running distance within each interval was reduced by approximately 2–3% for every change of direction in order to offset the greater energetic demands associated with multidirectional movement compared to straight-line running while maintaining an appropriate energy balance across the session. Overall training load was quantified using arbitrary training units (ATUs). To be considered eligible for inclusion in the experimental program, participants were required to complete a minimum of 80% of the scheduled training sessions [23]. Training adherence was recorded for each participant, and only athletes who fully met the compliance criterion were included in the final analysis.

The control group maintained their regular basketball training regimen, which included standard conditioning, technical, and tactical training, without participation in the HIIT intervention. This protocol ensured exercise was performed at near-maximal intensity, targeting both aerobic and anaerobic energy systems.

### 2.5. Statistical Analysis

Analyses were conducted on a per-protocol basis, including only participants who completed the post-intervention testing. Data normality was assessed using the Shapiro–Wilk test, while homogeneity of variances was examined with Levene’s test. Descriptive statistics are presented as mean ± standard deviation (M ± SD) together with 95% confidence intervals (95% CI). A two-way repeated-measures ANOVA was applied to evaluate the effects of the between-subjects factor Group (HIIT vs. CON) and within-subjects factor Time (pre- vs. post-intervention), as well as the interaction effect Group × Time. In cases where a significant interaction effect was observed, a Sheffé post hoc analysis was applied to determine pairwise differences. Effect size magnitude was assessed using partial eta squared (ηp^2^), classified through thresholds as small (0.01), medium (0.06), and large (0.14). Statistical significance was established at *p* < 0.05. All data were analyzed using the statistical software package STATISTICA 14 (Cloud Software Group, Inc.—Palo Alto, CA, USA (2023), Data Science Workbench).

## 3. Results

Table 2 shows that the HIIT group exhibited pre-to-post improvements in most physical performance variables from pre- to post-testing, whereas the control group exhibited only minor changes. Specifically, the HIIT group achieved faster sprint times across all measured distances (5 m, 10 m, and 20 m); improved agility performance (505, Pro-agility, and Zig-zag); enhanced repeated sprint ability (RSA mean and RSA best); and greater explosive power through improvements in CMJ, CMJA, and SJ performance following the intervention. In addition, substantial improvements were observed in aerobic performance variables, including VIFT and VO_2_max. Body mass and BMI slightly decreased in the HIIT group, while the control group did not demonstrate significant differences between the initial and final measurements in the analyzed variables. Overall, these findings suggest that the HIIT intervention positively affected aerobic fitness, sprint ability, agility, repeated sprint performance, and explosive power in female basketball players.

Table 3 confirms significant main effects of Treatment for most performance variables, including RSA mean (*p* = 0.001, η^2^ = 0.351), RSA best (*p* = 0.001, η^2^ = 0.324), VIFT (*p* < 0.001, η^2^ = 0.678), VO_2_max (*p* < 0.001, η^2^ = 0.840), 5 m sprint (*p* < 0.001, η^2^ = 0.452), 10 m sprint (*p* = 0.035, η^2^ = 0.155), 505 agility test (*p* = 0.009, η^2^ = 0.229), Pro-agility R (*p* < 0.001, η^2^ = 0.416), Zig-zag (*p* < 0.001, η^2^ = 0.423), CMJ (*p* < 0.001, η^2^ = 0.473), CMJA (*p* < 0.001, η^2^ = 0.686), and SJ (*p* < 0.001, η^2^ = 0.681). Significant main effects of Group were observed for BMI (*p* = 0.008, η^2^ = 0.231), VO_2_max (*p* < 0.001, η^2^ = 0.871), Pro-agility R (*p* = 0.001, η^2^ = 0.366), Pro-agility L (*p* = 0.003, η^2^ = 0.278), and CMJA (*p* = 0.008, η^2^ = 0.234), suggesting differences between the HIIT and control groups in these variables.

Furthermore, significant Group × Time interaction effects were identified for body mass (*p* = 0.003, η^2^ = 0.290), BMI (*p* = 0.005, η^2^ = 0.257), VIFT (*p* < 0.001, η^2^ = 0.511), VO_2_max (*p* < 0.001, η^2^ = 0.818), 5 m sprint (*p* < 0.001, η^2^ = 0.514), 10 m sprint (*p* = 0.013, η^2^ = 0.208), 20 m sprint (*p* = 0.011, η^2^ = 0.214), and Zig-zag agility test (*p* < 0.001, η^2^ = 0.423), indicating that the HIIT intervention produced greater improvements compared with the control condition. The largest interaction effects were observed for VO_2_max, VIFT, and 5 m sprint performance. In contrast, no significant interaction effects were observed for RSA variables, the 505 agility test, Pro-agility tests, CMJ, CMJA, and SJ, indicating similar changes between groups in these measures.

**Figure 1:**
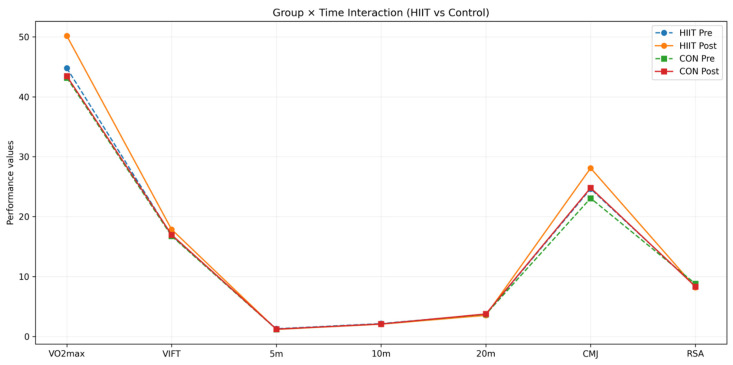
Group × Time interaction (HIIT vs. CON).

**Figure 2:**
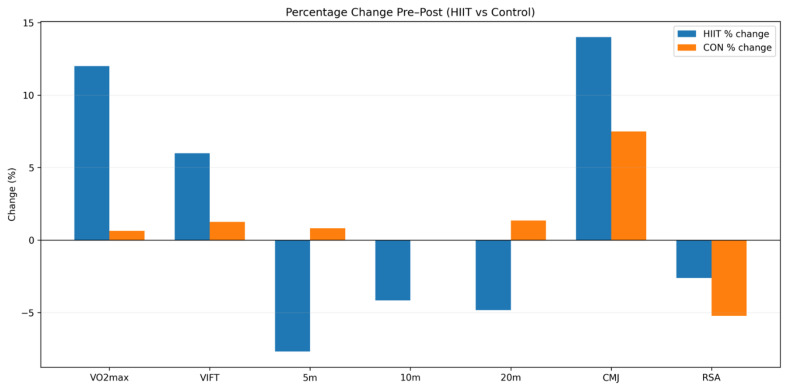
Percentage change pre–post.

Figure 1 illustrates the Group × Time interaction between the HIIT and CON groups, whereas Figure 2 displays the between-group differences in percentage change from pre- to post-intervention.

## 4. Discussion

The aim of this study was to examine the effects of a six-week high-intensity interval training (HIIT) on aerobic–anaerobic fitness, sprint performance, agility, repeated sprint ability, and explosive power in elite female basketball players. The main findings indicate that the implemented HIIT produced significant positive adaptations in several key components of physical fitness compared with the control group following the intervention. Significant Group × Time interaction effects were observed for body mass, BMI, VIFT, VO_2_max, 5 m sprint, 10 m sprint, 20 m sprint, and the Zig-zag agility test, with VO_2_max and VIFT representing the most prominent training-induced adaptations. These results should be interpreted primarily based on Group × Time interactions, as significant main effects of time likely reflect general training adaptations in both groups.

The sprint test results demonstrated significant improvements in running speed over short distances, particularly in the 5 m and 10 m sprints, where significant treatment and Group × Time interaction effects were observed. A similar pattern of greater improvements in shorter sprint distances compared with longer sprints has been reported in studies employing HIIT and sprint interval training protocols in female basketball players, where improvements in 10 m sprint performance reached approximately 7.2%, whereas improvements in 20 m sprint performance were around 3.2% [7,11]. These improvements may be attributed to neuromuscular adaptations induced by high-intensity training, including enhanced motor unit recruitment, increased explosive force production, and more efficient force generation during the initial phases of sprinting, as supported by review studies examining the role of HIIT in the development of strength and explosiveness [24,25,26,27]. Regarding agility, the results revealed an overall improvement in 5–0–5 test performance, indicating that both groups progressed throughout the intervention period. This finding may be explained by the fact that basketball inherently involves a large number of accelerations, decelerations, and changes of direction. The Pro-agility test results further revealed an asymmetric response between the dominant and non-dominant sides, which may indicate the presence of lateral differences in training adaptations.

The results of this study also indicate improvements in explosive power, confirming the positive influence of HIIT on neuromuscular performance. These findings are consistent with those reported by Mourgan et al. [28], who demonstrated that a five-week basketball-specific HIIT significantly enhanced lower-limb explosive power in young female basketball players. Following 12 training sessions, significant improvements were observed in both vertical jump and standing long jump performance. Similar findings were reported by Haghighi et al. [11], who found that a six-week HIIT performed twice weekly significantly increased jump-specific power in young female basketball players, with substantial improvements compared to the control group (ηp^2^ = 0.372–0.555). Likewise, Stanković et al. [25] reported that both HIIT and small-sided game (SSG) training produced significant improvements in explosive power assessed through CMJ, CMJ with arm swing, and SJ tests, without significant differences between the applied training models. Furthermore, the systematic review by Čaprić et al. [8] demonstrated that four to twelve weeks of HIIT training in female basketball players resulted in improvements of approximately 5–10% in CMJ and CMJa performance, while SJ performance improved by approximately 10–15%. From a broader perspective, the meta-analysis conducted by Wang et al. [29] confirmed moderate to large positive effects of HIIT on jumping performance across various sports, regardless of athletes’ sex.

Significant improvements were also observed in VIFT and VO_2_max, with large effect sizes compared to the control group. Similar results were reported by Mourgan et al. [28], who found that a five-week basketball-specific HIIT performed at 90–95% of maximal heart rate significantly increased VO_2_max in young female basketball players, with an overall improvement of approximately 5.6%. Comparable findings were reported by Aschendorf et al. [9] and Deak et al. [10], who demonstrated that basketball-specific HIIT lasting five to six weeks significantly improved aerobic endurance. In addition, Berisha et al. [30] showed that even a short-term ten-day HIIT can induce significant improvements in intermittent endurance among U16 female basketball players. Similar physiological adaptations have also been reported in female athletes participating in other team sports, where HIIT significantly improved VO_2_max and VIFT, resulting in practically meaningful enhancements in sport performance [31,32].

Our findings regarding the significant increase in VIFT are further supported by the results of Sanchez-Snchez et al. [33], who demonstrated that a six-week HIIT incorporating three changes of direction resulted in greater improvements in VIFT and repeated sprint ability (RSA) compared with a protocol involving only one change of direction. However, no superior effects of HIIT were observed for repeated sprint ability compared with the control group. This interpretation is supported by the findings of Stanković et al. [25], who reported statistically significant improvements across all RSA variables, including reductions in fatigue index, without significant differences between experimental groups. This interpretation is further supported by the study of Zeng et al. [13], which reported significant improvements in RSA performance following both small-sided games and HIIT with changes of direction, without a clear advantage of either training model. Similarly, Stanković et al. [34] noted that most HIIT studies involving female athletes reported improvements in RSA performance, whereas Li et al. [35] highlighted that small-sided game training can also induce positive adaptations in RSA, although the effects are often more variable than those observed following HIIT. The increase in VIFT values indicates an enhanced capacity to perform repeated high-intensity efforts interspersed with short recovery periods, which represents one of the fundamental physiological demands of modern basketball. Therefore, the present findings further support the growing body of evidence indicating that HIIT is among the most effective conditioning methods for improving aerobic capacity, sport-specific endurance, and the ability to maintain high-intensity activity throughout competition [7,8]. These findings may be useful for strength and conditioning coaches working with elite female basketball players during the preparatory period.

Although the results of this study demonstrated that a six-week high-intensity interval training program can significantly improve physical fitness, several limitations should be considered when interpreting the findings. First, aerobic capacity was assessed using the 30–15 Intermittent Fitness Test, and VO_2_max values were estimated rather than directly measured through laboratory procedures. Although this test is considered a reliable indicator of intermittent endurance, performance may be influenced by factors such as motivation, testing conditions, and individual test approach.

Furthermore, adaptations to the applied HIIT were not solely attributable to the intervention, as factors such as sleep, recovery, stress levels, and energy availability were not systematically monitored. In addition, external and internal training loads outside planned sessions were not fully controlled, which may have influenced individual responses. Another limitation is the absence of physiological and biochemical markers, which would provide deeper insight into adaptation mechanisms.

A further limitation is the relatively small sample size, typical for elite athlete studies, which may limit generalizability. All participants were elite female basketball players, so findings cannot be directly applied to other populations. The six-week duration was sufficient for initial adaptations but not for assessing long-term effects, and no follow-up measurements were conducted. Additionally, dietary habits and external activities were not fully controlled. These findings provide practical guidance for strength and conditioning coaches in women’s basketball, indicating that HIIT should be prioritized for improving aerobic and intermittent performance, while RSA and explosive power may require additional specific training methods.

Future research should consider longer interventions, larger samples, and longitudinal follow-ups. More comprehensive monitoring of recovery, nutrition, training load, and physiological markers would further improve understanding of HIIT adaptations in female basketball players.

## 5. Conclusions

The results of this study indicate that a six-week HIIT is an effective method for improving aerobic fitness, sprint performance, and change-of-direction ability in elite female basketball players. Significant Group × Time interaction effects were observed for VO_2_max, VIFT, sprint performance (5 m, 10 m, and 20 m), and Zig-zag agility performance, indicating greater improvements compared with the control group. The most pronounced effects were observed in aerobic and intermittent performance capacities (VO_2_max and VIFT), highlighting the primary adaptations to the applied HIIT protocol. These findings confirm that HIIT represents a practical and time-efficient conditioning method for enhancing physical fitness and performance in female basketball players. No superior effects of HIIT were observed for repeated sprint ability or jumping performance compared with the control group, suggesting that these abilities may require more specific or additional training stimuli. Future research should examine the long-term effects of different HIIT protocols and their impact on basketball-specific performance indicators, with particular emphasis on practical applications for strength and conditioning coaches working in women’s basketball.

## Figures and Tables

**Table 1:** HIIT training program.

Week	Intensity (%) (Work/Rest)	Repetition	Series	Duration (s)	Training Load (ATU)
1–2	100	8	3	15	18.000
3–4	100	10	3	15	22.500
5–6	100	12	3	15	27.000

**Table 2:** Descriptive statistics: mean ± standard deviation (M ± SD), 95% confidence interval (95% CI).

	HIIT		CON	
	Initial	Final	Initial	Final
RSA mean	8.43 ± 0.27 8.29–8.58	8.21 ± 0.21 ^a^ 8.10–8.33	8.81 ± 0.68 8.40–9.22	8.35 ± 0.40 ^b^ 8.11–8.59
RSA best	8.13 ± 0.32 7.96–8.30	7.99 ± 0.19 ^a^ 7.89–8.09	8.48 ± 0.59 8.12–8.83	8.08 ± 0.37 ^b^ 7.86–8.31
VIFT	16.86 ± 0.82 16.42–17.30	17.87 ± 0.75 ^a^ 17.47–18.27	16.77 ± 0.63 16.37–17.17	16.98 ± 0.53 16.66–17.30
Vo_2_max	44.77 ± 2.23 43.58–45.96	50.14 ± 1.49 ^a^ 49.35–50.93	43.16 ± 0.88 42.56–43.73	43.43 ± 0.89 42.81–44.05
5 m	1.30 ± 0.09 1.26–1.35	1.20 ± 0.07 ^a^ 1.16–1.24	1.23 ± 0.05 1.20–1.27	1.24 ± 0.06 1.21–1.27
10 m	2.16 ± 0.11 2.10–2.22	2.07 ± 0.11 ^a^ 2.01–2.13	2.10 ± 0.10 2.04–2.15	2.10 ± 0.10 2.04–2.17
20 m	3.72 ± 0.16 3.63–3.80	3.54 ± 0.10 ^a^ 3.48–3.59	3.72 ± 0.23 3.58–3.86	3.77 ± 0.32 3.58–3.96
505	2.63 ± 0.39 2.42–2.84	2.49 ± 0.13 ^a^ 2.42–2.56	2.64 ± 0.17 2.54–2.75	2.51 ± 0.10 ^b^ 2.46–2.57
Pro-agility R	5.39 ± 0.20 5.28–5.50	5.15 ± 0.25 ^a^ 5.02–5.28	5.72 ± 0.32 5.52–5.91	5.55 ± 0.33 ^b^ 5.35–5.75
Pro-agility L	5.36 ± 0.25 5.23–5.50	5.28 ± 0.34 5.10–5.46	5.69 ± 0.37 5.47–5.92	5.57 ± 0.29 5.39–5.75
Zig Zag	6.36 ± 0.33 6.19–6.53	6.17 ± 0.24 ^a^ 6.04–6.30	6.50 ± 0.45 6.23–6.77	6.50 ± 0.45 6.23–6.77
CMJ	24.65 ± 2.67 23.23–26.07	28.10 ± 3.84 ^a^ 26.05–30.15	23.08 ± 3.83 20.77–25.40	24.81 ± 5.19 ^b^ 21.67–27.94
CMJA	28.41 ± 2.54 27.05–29.76	33.43 ± 4.42 ^a^ 31.07–35.78	24.90 ± 4.51 22.18–27.62	28.45 ± 5.25 ^b^ 25.28–31.63
SJ	22.28 ± 2.31 21.04–23.51	26.64 ± 3.02 ^a^ 25.03–28.25	19.79 ± 5.40 16.53–23.05	23.72 ± 5.47 ^b^ 20.42 ± 27.03

0–10, 0–20, 0–30—running test; CMJ—countermovement jump; CMJA—countermovement jump with free arms; SJ—squat jump; RSA—repeated speed agility; VIFT—final speed achieved in the 30–15 Intermittent Fitness Test; ^a^ significant difference from initial measurement in the HIIT group; ^b^ significant differences from initial measurement in the CON group.

**Table 3:** Two-way repeated-measures ANOVA results for the Treatment × Group interaction. Main effect of between-subjects factor Group and within-subjects factor Treatment. F value (F), statistical significance (*p*), partial eta squared (ηp2).

	Group			Treatment			Interaction		
	F	*p*	η^2^	F	*p*	η^2^	F	*p*	η^2^
RSA mean	4.207	0.050	0.135	14.630	0.001	0.351	1.815	0.189	0.063
RSA best	3.175	0.086	0.105	12.919	0.001	0.324	2.819	0.105	0.095
VIFT	3.860	0.060	0.129	54.655	<0.001	0.678	27.185	<0.001	0.511
Vo_2_Max	175.343	<0.001	0.871	136.843	<0.001	0.840	117.160	<0.001	0.818
5 m	0.430	0.518	0.016	22.260	<0.001	0.452	28.526	<0.001	0.514
10 m	0.176	0.678	0.006	4.954	0.035	0.155	7.080	0.013	0.208
20 m	3.257	0.082	0.108	1.956	0.173	0.068	7.358	0.011	0.214
505	0.070	0.794	0.003	8.021	0.009	0.229	0.002	0.968	0.000
Pro-agility R	15.556	0.001	0.366	19.261	0.000	0.416	0.639	0.431	0.023
Pro-agility L	10.420	0.003	0.278	2.310	0.140	0.079	0.119	0.733	0.004
Zig Zag	2.945	0.098	0.098	19.759	<0.001	0.423	19.759	<0.001	0.423
CMJ	3.172	0.086	0.105	24.207	<0.001	0.473	2.698	0.112	0.091
CMJA	8.241	0.008	0.234	59.078	<0.001	0.686	1.725	0.200	0.060
SJ	3.482	0.073	0.114	57.548	<0.001	0.681	0.156	0.696	0.006

Legend: 0–5, 0–10, 0–20—running test; CMJ—countermovement jump; CMJA—countermovement jump with free arms; SJ—squat jump; RSA—repeated speed agility.

## Data Availability

The original contributions presented in this study are included in the article. Further inquiries can be directed to the corresponding author.
